# Importance of Crime Scene Visits by a Forensic Medicine Expert: A Survey-Based Study

**DOI:** 10.7759/cureus.26775

**Published:** 2022-07-12

**Authors:** Binay Kumar, Nilabh P Singh, Nawal Singh, Nikhil Goel

**Affiliations:** 1 Forensic Medicine & Toxicology, All India Institute of Medical Sciences, Patna, Patna, IND

**Keywords:** coroner, magistrate, police, forensic medicine expert, medical examiner, inquest, crime investigation, crime scene

## Abstract

Background

This study is intended to compare the Indian system of inquests, which is conducted by nonscientific people like police and magistrate (who are compulsorily neither qualified in science nor in law), and the medical examiner system of the USA which is done by doctors specialized in forensic medicine/pathology.

Aims

The goal of this study was to see if bringing in a medical examiner system makes a difference in determining the cause, manner, and time of death as compared to the current system of conducting inquests and autopsies by two different agencies, namely, the police and forensic medicine experts.

Material and methods

In the present study, a peculiar case (in which the police were clueless and the autopsy surgeon was confused during the autopsy) was chosen for getting an expert opinion from 50 forensic medicine experts from different parts of the country in which police were not clear regarding cause, manner and time of death, but later after a crime scene visit by forensic medicine experts who had conducted the autopsy, it was clarified. Opinion regarding the cause, manner, and time since death was taken from 50 medico-legal experts in two steps. In the first step, only the autopsy finding and history obtained from police were provided and in the second step, additional information obtained from the crime scene visit by forensic medicine experts was provided.

The sampling method was purposive sampling.

Result

In the cause of death, Cohen’s Kappa coefficient was 0.30% (z-statistic = 3.87, p-value = 0.0001), indicating a significantly low agreement between the first and second steps by the experts, as their decision changed after getting the evidence of the crime scene visit. In the manner of death, Cohen’s Kappa coefficient was 0.06% (z-statistic = 0.66, p-value = 0.2540) indicating a very low agreement between the first and second opinions by the experts as their decision regarding the suspected manner of death changed drastically after getting the evidence of crime scene. In the time since death, Cohen’s Kappa coefficient was 0.5531% (z-statistic = 7.25, p-value = 0.0001), which also indicates significant difference.

Conclusion

Therefore, in this study, the usefulness of the medical examiner system in the Indian setting has been proved beyond doubt expecting drastic improvement in criminal investigation by introducing the medical examiner system in India.

## Introduction

Medicine and law have been linked in Egyptian society since 3000 B.C. [1]. The role of the medical man has been recognized at almost every stage of the crime/death investigation and trial, including body examination and evidence collection, history, interviewing witnesses, physical examination, autopsy examination, laboratory test, court depositions, and their interpretation and collaboration [1-2]. In the present day, several crime investigation procedures exist in many countries. Every system has advantages and disadvantages. As a result, there are debates taking place in various regions of the world to build a better system of criminal investigations.

Inquest is the term used to describe the initial investigation at the scene of the incident. The legal inquiry into the cause of death is known as an inquest. When information about a dead human body found in unnatural or suspicious circumstances reaches the authority authorized to hold an inquest, the concerned authority travels to the scene of the incident and conducts an investigation, after which they prepare an inquest report detailing the apparent cause and circumstances of death. The body is then sent to the nearest authorized autopsy center if an autopsy was required. Among the numerous systems, the following are important inquest systems [3-5]: police inquest, magistrate inquest, coroner system of inquests, medical examiner system of inquests, and jury system.

Only police and magistrate inquests are conducted in India under sections 174 and 176 Cr.P.C., respectively, and they are public servants with limited exposure to law and forensic science throughout their professional training, but they are scientifically lay individuals [6-7]. In India, the crime scene investigation is handled by a different agency while autopsy and other scientific investigations are handled by a different agency. And all of these agencies are functioning in isolation, with minimal coordination. Coroners, on the other hand, are elected officials in the United States who may or may not be qualified in medicine or law. However, under British control, India too had a coroner system, which was gradually supplanted by the police and magistrate system after independence and was eventually abolished on July 29, 1999, in Mumbai [8]. Coroners in India used to be qualified in either law or medicine or both. In 1857, Dr. Urquhart, a private practitioner and coroner of the city, was appointed as the first Professor of Forensic Medicine at Madras Medical College [3-5,9] coroners in India used to be the status of first-class magistrates [5]. The medical examiner system of inquests is widely used in the United States, Canada, and Japan, and medical examiners are trained in both medicine (forensic pathology) and law. This is widely regarded as the best inquest system in the world [3-5]. When the same individual visits the crime scene for an inquest, conducts an autopsy, and correlates laboratory findings, they are better able to correlate because they are both medically and legally qualified.

## Materials and methods

Aim and objective of the study

The goal of this study was to see if bringing in a medical examiner system makes a difference in determining the cause, manner, and time of death as compared to the current system of conducting inquests and autopsies by two different agencies, namely, police and forensic medicine expert in India.

Methodology

In this study, the forensic medicine specialist who performed the autopsy also went to the crime scene and interviewed witnesses, drawing conclusions about the cause, manner, and time of death that the police investigation had not revealed. In the first step, autopsy findings and information received from the police during the autopsy were sent to 102 forensic medicine professionals on their WhatsApp contact numbers. They were then asked to comment on the cause, manner, and time of death by designing multiple-choice questions through a Google link. Out of them, 79 participants responded. Following that, the new information gathered by visiting the crime scene and questioning witnesses by the autopsy surgeon was shared with those 79 participants for a second time, and their opinions on the matter were sought once more. The opinions of the first 50 participants who responded the second time were included in the analysis. Thereafter, the results were evaluated to see how new information affects expert opinions through the same questions. The study was approved by the Institutional Research Committee vide ref. no AIIMS/Pat/IRC/2020/835, dated: 04/12/2021, and the Institutional Ethics Committee vide ref. no AIIMS/Pat/IEC/2020/835, dated: 04/12/2021, of All India Institute of Medical Sciences, Patna.

Sampling Method

Purposive sampling was used. The contact numbers of participants were obtained from various WhatsApp groups of forensic medicine experts in India. In the current study, most of the forensic medicine experts included are working in the department of forensic medicine & toxicology of reputed government medical institutions in India; however, a few are working in private medical colleges also. As previous such studies could not be found, the exact sample size was not calculated.

Statistical Analysis

After correct coding, all the data were loaded into Microsoft Excel 2019 (Microsoft Corporation, Redmond, WA). SPSS version 22 (IBM Corp., Redmond, WA) was used for the statistical analysis. The proportions of categorical variables were used. The chi-square test was used to determine the relationship between two categorical variables. On the first and second attempts, Cohen's Kappa coefficient was calculated to determine the extent of expert agreement.

## Results

**Figure 1:**
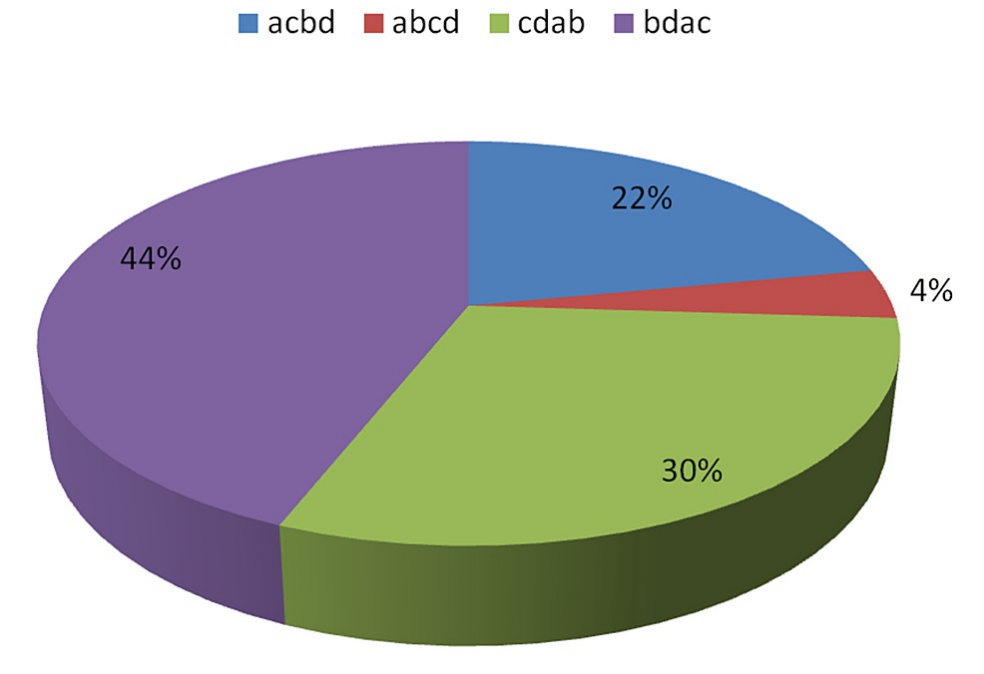
Suspected cause of death in decreasing order of probability in the first step a = head injury, b = homicidal strangulation, c = accidental traumatic asphyxia, d = spinal cord injury

**Figure 2:**
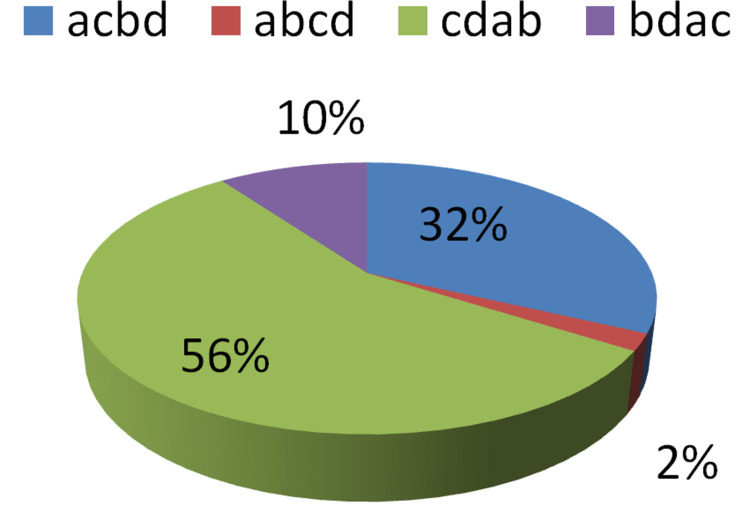
Suspected cause of death in decreasing order of probability in the second step a = head injury, b = homicidal strangulation, c = accidental traumatic asphyxia, d = spinal cord injury

In response to questions on the cause-of-death opinions, it was observed that in the first phase, when just autopsy findings and information accessible from police at the time of autopsy were presented, only 30% of participants offered the right answers and 70% gave incorrect answers. After presenting further information gathered from a crime scene visit and questioning witnesses by forensic medicine specialists who conducted an autopsy in the second stage, 56% of experts gave correct opinions and 44% supplied incorrect opinions. Table 1 contains information on various replies, which is illustrated in Figures 1-2. The percent agreement was calculated as only 50%. Cohen's Kappa coefficient was 0.30% (z-statistic = 3.87, p-value = 0.0001), revealing a considerable lack of agreement between the experts in the first and second steps, as their decision improved after seeing the evidence at the crime scene.

**Table 1:** Opinion received regarding the suspected cause of death in decreasing order of probability in Step I and Step II a = head injury, b = homicidal strangulation, c = accidental traumatic asphyxia, d = spinal cord injury Pearson chi2 (9) = 49.1872; Fisher's exact = 0.000

Opinion regarding the suspected cause of death in decreasing order of probability in the first step			Opinion regarding the suspected cause of death in decreasing order of probability in the second step	
Responses	Number	Percent	Number	Percent
acbd	11	22%	16	32%
abcd	2	4%	1	2%
cdab	15	30%	28	56%
bdac	22	44%	5	10%
Correct	15	30%	28	56%
Incorrect	35	70%	22	44%

**Figure 3:**
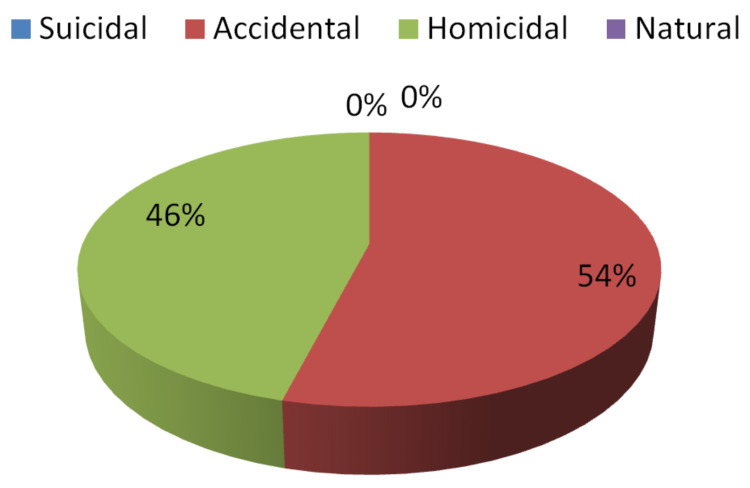
Opinion regarding the manner of death in Step I

**Figure 4:**
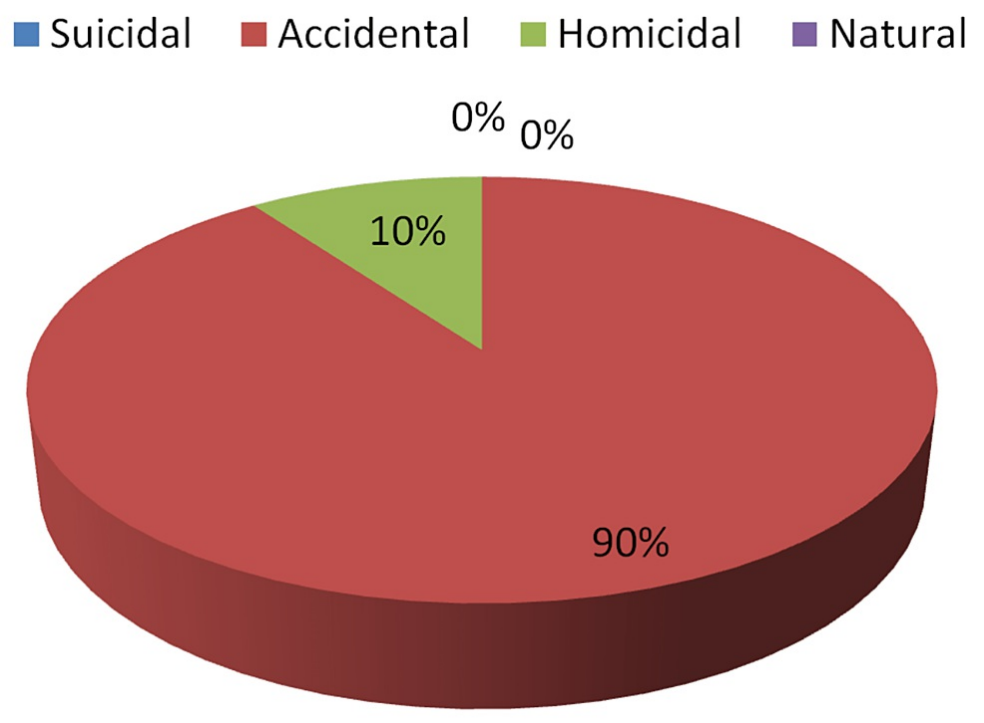
Opinion regarding the manner of death in Step II

Only 54% of specialists were able to correctly answer questions about the manner of death in the first phase while 90% were able to do so in the second. In terms of statistics, the percent agreement was calculated as 56% with a Cohen's Kappa coefficient of 0.06% (z-statistic = 0.66, p-value = 0.2540), indicating a very low agreement between the experts' first and second attempts as their decision regarding the suspected manner of death changed dramatically after receiving evidence of crime location (Table 2) contains information on various responses, which is depicted in Figures 3-4.

**Table 2:** Opinion received regarding the suspected manner of death in Step I and Step II

Opinion regarding the suspected manner of death in the first step			Opinion regarding the suspected manner of death in the first step	
Responses	Number	Percent	Number	Percent
Suicidal	0	0%	0	0%
Accidental	27	54%	45	90%
Homicidal	23	46%	5	10%
Natural	0	0%	0	0%
Correct	27	54%	45	90%
Incorrect	23	46%	5	10%
Pearson chi2 (1) = 0.4384; Fisher's exact = 0.651				

**Figure 5:**
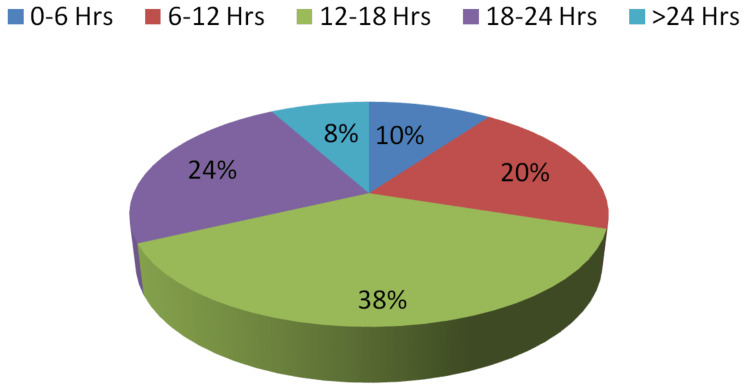
Opinion regarding time since death in Step I

**Figure 6:**
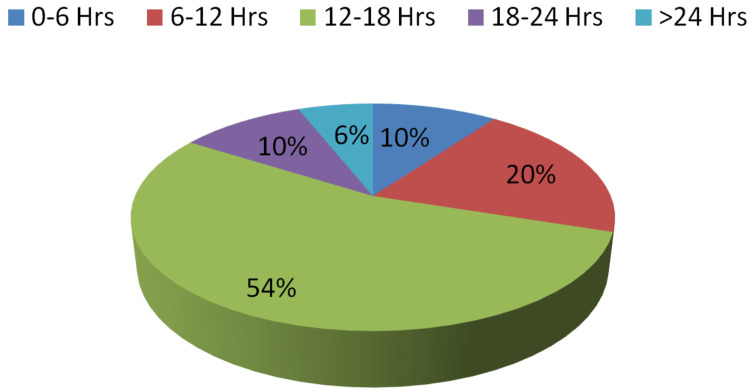
Opinion regarding time since death in Step II

Regarding time since death in the first phase, 38% of experts gave the correct response, whereas 54% supplied the correct answer in the second step. Percent agreement was found to be 68% with Cohen's Kappa coefficient of 0.5531 (z-statistic = 7.25, p-value = 0.0001). However, the difference of opinion is less in comparison to the other two opinions but is significant. Table 3 contains information on various responses, which is depicted in Figures 5-6.

**Table 3:** Opinion received regarding time since death in Step I and Step II

Opinion regarding time since death in Step I			Opinion regarding time since death in Step II	
Responses	Number	Percent	Number	Percent
0-6 Hrs	5	10%	5	10%
6-12 Hrs	10	20%	10	20%
12-18 Hrs	19	38%	27	54%
18-24 Hrs	12	24%	5	10%
>24 Hrs	4	8%	3	6%
Correct	19	38%	27	54%
Incorrect	31	62%	23	46%
Pearson chi2 (16) = 88.2084; Fisher's exact = 0.000				

## Discussion

The main difference between the medical examiner system and other inquest systems is that in the medical examiner system, a significant portion of the criminal investigation, such as body examination and evidence collection at the scene of the crime, history, interviewing witnesses, physical examination, autopsy examination, laboratory test, toxicology, and their interpretation and collaboration, and the deposition in courts, is done or supervised by the same person, i.e. the medical examiner [10-11]. In other systems of the inquest, the job of the medical man is confined to the preparation of autopsy and injury reports, with the rest of the work such as the interpretation and collaboration of scientific studies, being handled by scientifically lay persons. In India, the situation of such highly specialized and expert jobs requiring painstaking work is the worst, and the lowest ranking police officers, such as assistant sub-inspectors or head constables, are frequently assigned for this. In every inquest, the initial professional assessment of the body and its surroundings at the scene of the crime is critical [2].

The current study clearly shows that, as is common in the medical examiner system, when a forensic medicine specialist visits the crime scene, interviews witnesses, and interprets the evidence, a greater picture of the incident's circumstances is gained. The fact that the conviction rate in Federal Court in the United States, where a medical examiner system is in place, is around 99.8% [12], whereas it is about 50.4% in India, demonstrates this [13].

To assess the system, a committee was formed in 2003 in Washington (DC) to organize a workshop on the Medicolegal Death Investigation System, and after the workshop, the following important recommendations or observations were given in the workshop summary listed in Table 4 [11].

**Table 4:** Recommendation/observations of the committee formed in 2003 in Washington (DC) to organize a workshop on the medicolegal death investigation system

Sl. No.	Recommendations/Observations
1	Death certification should be performed by highly qualified medical specialists who are capable of collaborating autopsy findings with the crime scene and laboratory results, as well as examining immediate and prior medical histories, interviewing witnesses, and doing physical examinations.
2	An ideal medical examiner system would be led by medical schools, which have subspecialty facilities, such as pathology, forensic science laboratories, public health systems, and laboratories.
3	Being an elected system, there are two major drawbacks to the coroner system: one is that the coroners are less likely to be medically competent and the other is the inadequate legislative system.
4	Finally, at the workshop, it was determined to promote the abolition of the coroner system on a nationwide level in order to establish higher professionalism through the medical examiner system.

Based on different circumstances and experiences, the coroner system in Washoe County was totally replaced by the medical examiner system by County Ordinance in 2007, and the Chief Medical Examiner was given the title of Coroner [11].

Limitations of the study

In this study, an interview was conducted only among forensic medicine experts. Magistrates and police officers who are currently conducting inquests should have been included in the study too for better comparison among different systems.

## Conclusions

It is widely acknowledged that the medical examiner system is the most effective method of death investigation, in which the medical examiner performs a variety of tasks such as crime scene investigation, evidence collection, witness interviews, autopsy examination, and laboratory result interpretation. The findings of this study backed up the general consensus. Medical examiners must have a postgraduate or doctorate degree in forensic medicine or forensic pathology, as well as the necessary legal training. The system should be overseen and directed by the forensic medicine section of the medical institution of a particular region.
